# Young COVID-19 Patients Show a Higher Degree of Microglial Activation When Compared to Controls

**DOI:** 10.3389/fneur.2022.908081

**Published:** 2022-06-16

**Authors:** Jakob Matschke, Henri Lahann, Susanne Krasemann, Hermann Altmeppen, Susanne Pfefferle, Giovanna Galliciotti, Antonia Fitzek, Jan-Peter Sperhake, Benjamin Ondruschka, Miriam Busch, Natalie Rotermund, Kristina Schulz, Christian Lohr, Matthias Dottermusch, Markus Glatzel

**Affiliations:** ^1^Institute of Neuropathology, University Medical Center Hamburg-Eppendorf, Hamburg, Germany; ^2^Institute of Medical Microbiology, University Medical Center Hamburg-Eppendorf, Hamburg, Germany; ^3^Institute of Legal Medicine, University Medical Center Hamburg-Eppendorf, Hamburg, Germany; ^4^Institute of Cell and Systems Biology of Animals, University of Hamburg, Hamburg, Germany

**Keywords:** SARS-CoV-2, COVID-19, nervous system, neuropathology, neuroinflammation, microglia, astrocytes

## Abstract

The severe acute respiratory syndrome-corona virus type 2 (SARS-CoV-2) is the cause of human coronavirus disease 2019 (COVID-19). Since its identification in late 2019 SARS-CoV-2 has spread rapidly around the world creating a global pandemic. Although considered mainly a respiratory disease, COVID-19 also encompasses a variety of neuropsychiatric symptoms. How infection with SARS-CoV-2 leads to brain damage has remained largely elusive so far. In particular, it has remained unclear, whether signs of immune cell and / or innate immune and reactive astrogliosis are due to direct effects of the virus or may be an expression of a non-specific reaction of the brain to a severe life-threatening disease with a considerable proportion of patients requiring intensive care and invasive ventilation activation. Therefore, we designed a case-control-study of ten patients who died of COVID-19 and ten age-matched non-COVID-19-controls to quantitatively assess microglial and astroglial response. To minimize possible effects of severe systemic inflammation and / or invasive therapeutic measures we included only patients without any clinical or pathomorphological indication of sepsis and who had not been subjected to invasive intensive care treatment. Our results show a significantly higher degree of microglia activation in younger COVID-19 patients, while the difference was less and not significant for older COVID-19 patients. The difference in the degree of reactive gliosis increased with age but was not influenced by COVID-19. These preliminary data warrants further investigation of larger patient cohorts using additional immunohistochemical markers for different microglial phenotypes.

## Introduction

After emerging from the city of Wuhan in China in early December 2019, infections with the novel severe acute respiratory syndrome-corona virus type 2 (SARS-CoV-2) have rapidly evolved into a global pandemic. SARS-CoV-2 is the cause for human coronavirus disease 2019 (COVID-19), a severe, life-threatening illness; as of March 2022, there have been more than 460 million cases worldwide, more than 6 million of which have been fatal (https://covid19.who.int/). COVID-19 is mainly a respiratory disease, yet neurological signs and symptoms such as stroke, seizures, and altered mental status have been reported in more than a third of patients during the acute phase of COVID-19 (1–3). Furthermore, about 10–30% of patients develop persistent symptoms and / or delayed or long-term complications following acute COVID-19; a syndrome including diverse neuropsychiatric sequelae, that has been called long COVID or post-acute COVID-19 (4). Thus, an understanding of the underlying pathophysiological mechanisms of CNS impairment is of paramount importance, which clearly shows the need for detailed neuropathological studies (5, 6). Indeed, neuropathological assessment has been proven as a valuable instrument for providing important insights into potential routes of virus neuroinvasion and mechanisms of CNS damage (7–11). A key question in the pathophysiology of COVID-19 in the CNS is the role of the neuroimmune system, in particular microglia cells and astrocytes. In fact, several studies report activation of the neuroimmune axis with microglial activation, presence of microglial nodules and context-specific interaction with CD8+ T cells (7, 12–14). Astrocytic activation is less studied in COVID-19, and it has been shown that older COVID-19 patients have more pronounced astrogliosis, although this is certainly influenced by age itself and / or by concomitant neurodegenerative conditions (14).

So far, many neuropathological autopsy studies of COVID-19 patients have had shortcomings due to the inherently high heterogeneity of the analyzed cohorts in terms of age, concomitant neurological diseases, and treatment regimens prior to death, since all these factors are known to influence both microglial and astroglial response (15–18). Therefore, we designed a case-control study of ten patients who died of COVID-19 and ten age-matched non-COVID-19 controls. Importantly, we only included patients who died either at home, in a nursery home, or in a hospital setting without intensive care treatment; furthermore, we excluded patients or controls with sepsis and / or any gross neuropathological findings considered to interfere with astroglial and / or microglial response (e.g., infarction, bleeding, tumor, trauma). Additionally, we stratified by age and analyzed a younger and an older age subgroup. We specifically studied the degree of lymphocytic infiltration and of both microglial and astroglial response by immunohistochemical staining and digital quantitative image analysis. Our results show a higher degree of microglia activation in both younger and older COVID-19 patients, being significantly different only for younger patients. The degree of reactive astrogliosis increased with age but was not found to be influenced by COVID-19 or the duration of the disease.

## Methods

### Autopsies and Ethical Considerations

In the federal state of Hamburg, Germany, all persons who had died due to or with an infection of SARS-CoV-2 during the period from 1 March to 31 December 2020 underwent full autopsy at the Institute of Legal Medicine of the University Medical Center of Hamburg-Eppendorf (UKE). SARS-CoV-2 infection was diagnosed using a throat swab followed by immediate RT-qPCR for viral SARS-CoV-2 RNA in all cases. The Ethics Committee of the Hamburg Chamber of Physicians was informed about the study (PV7311 and 2020-10353-BO-ff) and the study is in line with the Declaration of Helsinki. Clinical data including pre-existing medical conditions, medical course prior to death and ante mortem diagnostic findings were assessed. After removal during autopsy, all brains were fixed in buffered 4% formaldehyde, examined macroscopically, and underwent extensive neuropathological workup at the Institute of Neuropathology of the UKE. From the whole study cohort, cases were chosen by virtue of age, i.e., five patients from the youngest quartile (i.e., <60 years of age) and five patients from the oldest quartile (i.e., >90 years of age). Age-matched historical controls who died before the year 2019 were culled from the autopsy archive of the Institute of Neuropathology. Exclusion criteria for both cases and controls were pre-existing neurological disease (e.g., major cerebral infarction, tumor, intracranial bleeding, trauma), presence of sepsis according to clinical and / or pathological features, and invasive treatment regimens during intensive care. Since neurodegenerative changes are inevitably found in both COVID-19 patients and controls from the oldest quartile, this was not considered an exclusion criterion.

### Histopathological Examination

Formalin-fixed paraffin-embedded tissue (FFPE) samples from the superior frontal gyrus (Brodman area 9 or 8) were processed and stained with hematoxylin and eosin following standard laboratory procedures. Immunohistochemistry with antibodies to human glial fibrillary acidic protein (GFAP; 1:200, clone 6F2; DakoCytomation, Glostrup, Denmark), human leukocyte antigen DR, (HLA-DR, DP, DQ; 1:200, mouse clone CR3/43; DakoCytomation), CD8 (1:100, clone SP239; Spring Bioscience, Pleasanton, USA), and SARS-CoV-2 nucleocapsid (1:1000; clone 4A8, Synaptic Systems, Goettingen, Germany) was performed on a Ventana benchmark XT autostainer following the manufacturer's recommendations. The quality of the immunohistochemical stains was assessed by on-slide positive controls for all antibodies. For semiquantitative assessment of cytotoxic T lymphocyte infiltration, cells with positive CD8 staining were counted per high-power field (HPF) of 0.5 mm^2^. Infiltration was categorized as none, mild (1 to 9 cells per HPF), moderate (10 to 49 cells per HPF), or severe (≥50 cells per HPF).

### Immunohistochemistry on Free-Floating Sections

Blocks of paraffin-embedded cortical brain sections were cut into 200–250 μm thick slices and paraffin was removed with two washes in Roti-Histol (Carl Roth, Karlsruhe, Germany) at 36°C. Slices were rehydrated in a series of 100, 95, 70, and 50% ethanol (EtOH) and H_2_O. For antigen retrieval, slices were submerged in acidic buffer (10 mM citric acid, 0.05% Tween-20, pH 6.0) at 95°C for 10 min and then allowed to cool down to room temperature (RT) for 20 min. Slices were permeabilized in PBST (0.2% Triton-X100 in PBS) for 1 h at RT and then blocked for 1 h in 5% BSA, 0.4% Triton-X100 in PBS at room temperature (RT). Slices were incubated for 4–5 days with the following primary antibodies: mouse anti-GFAP (sc-33673, Santa Cruz Biotechnology, Heidelberg, Germany; 1:100) and rabbit anti-vimentin (GTX100619, GeneTex, Irvine, CA; 1:100) in 5% BSA, 0.2% Triton-X100, 0.1% sodium acid in PBS at 4°C. Slices were then incubated with secondary antibodies (goat anti-rabbit Alexa Fluor 488 and goat anti-mouse Alexa Fluor 555, Life Technologies, Darmstadt, Germany; 1:500 with 0.1% sodium acid in PBS) for 3–4 days at 4°C. Slices were covered with TrueBlack (Biozol, Eching, Germany; 1:20 in 70% EtOH) for 2 min to reduce autofluorescence and finally mounted in RapiClear 1.49 (SunJin Lab, Hsinchu City, Taiwan) followed by incubation for 30 min at RT and then analyzed with a confocal microscope (eC1, Nikon, Düsseldorf, Germany) using a 40x/NA 1.3 objective. Z-stacks were imaged with 0.15 μm axial spacing and deconvolved using Huygens software (SVI, Hilversum, Netherlands). Projections were produced with ImageJ (19) and adjusted for brightness and contrast with Photoshop (Adobe, Dublin, Ireland).

### Quantitative Image Analysis

**Figure 1:**
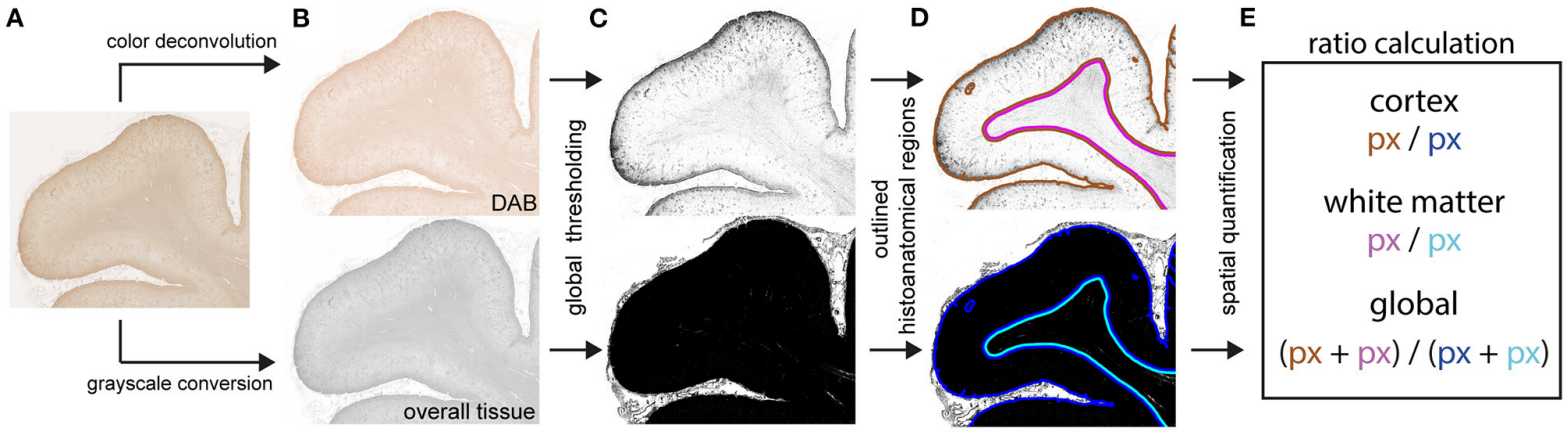
Schematic procedure for digitally supported quantification of immunostaining intensities. Digitalized slides of immunostained brain tissue **(A)** were separately deconvoluted for DAB [**(B)**, upper panel] and converted to grayscale [**(B)**, lower panel]. Both acquired binary images were globally thresholded **(C)** with distinct value cut-offs. Pixel quantities were measured within manually assigned anatomical regions **(D)**. Assessment of immunostaining intensities **(E)** was based on ratios calculated from pixels with above-threshold values of the deconvoluted DAB image [**(C)**, upper panel, DAB] over pixels with above-threshold value of the grayscale image [**(C)**, lower panel, overall tissue] within their respective anatomical regions **(D)**.

Immunohistochemically stained slides for both GFAP and HLA-DR/DP from COVID-19 patients and controls were digitalized using a Hamamatsu NanoZoomer 2.0-HT C9600 whole slide scanner (Hamamatsu Photonics, Tokyo, Japan). Images were exported using NDP view v2.7.43 software. Digital image analysis was performed using ImageJ/Fiji software (19). For measurement of the entire tissue area, whole slide images were first converted to grayscale and inverted. Then, normalization was achieved by subtraction of the mean gray value of representative, manually identified background pixels. Whole tissue areas were measured on re-inverted images *via* consistent global thresholding and subsequent pixel quantification. For measurement of positively stained areas, the analogous approach was implemented in the DAB color channel of whole slide images after utilization of the color deconvolution plugin. To account for comparable immunostaining quantification, the ratio of positively stained area (px) / entire tissue area (px) was calculated. For spatial immunostaining quantification, anatomically distinct areas (regions of interest, ROIs) were manually drawn and subjected to separate measurements analogous to the described approach (see Figure 1). Tissue areas, which were not eligible for quantification (e.g., due to technical or digital artifacts) were excluded from the analysis.

### Statistical Analysis

General demographic features were analyzed using standard methods of descriptive statistics. Since the study design was based on the comparison between case-control pairs, statistical analysis included the paired *t*-test for continuous variables of normal distribution (Welch's t-test modification was used if parameters showed unequal variance in Levene's test). For testing of normality, the Kolmogorov-Smirnov test was used. For the comparison of non-normally distributed continuous variables the Mann-Whitney-*U* test was applied. Frequency distributions were compared with binomial or χ2 tests. Significance was assumed with a *p* ≤ 0.05. Actual *p*-values were presented wherever possible, and the 95% confidence interval (95% CI) was calculated. Data were analyzed using IBM SPSS Statistics version 27.0.

## Results

### Demographic, Clinical, and General Neuropathologic Details of Cases and Controls

During the period from the 1 March to the 31 December 2020, there were 281 autopsies of patients who had died due to or with SARS-CoV-2 at the Institute of Legal Medicine of the University Medical Center Hamburg-Eppendorf (20). All brains of these autopsies had been removed and sent for detailed neuropathological analysis. Both from the youngest quartile (i.e., <60 years of age) and from the oldest quartile (i.e., >90 years of age) 5 cases each were chosen, provided they met the inclusion criteria (no preexisting neurological disease, no sepsis, and no invasive treatment regimens during intensive care). Disease duration was available for 7 out of 10 COVID-19 patients (range: 4–30 days; median = 14 days). Age-matched controls without exclusion criteria were then culled from the archive of the Institute of Neuropathology within a range of ±5 years. For demographic details of cases and controls (see Table 1). None of the COVID-19 patients and the controls showed any significant macroscopic damage to the brain (e.g., cerebral infarction, bleeding, tumor, trauma). Neither were there any microscopic findings that have been variously described for COVID-19 patients in the literature, e.g., demyelination, meningitis, encephalitis, endotheliitis, or widespread hyaline microthrombosis (21–23). Nine of 10 COVID-19 patients died of COVID-19 pneumonia and one of pulmonary embolism following deep venous thrombosis. For further details of cases and controls (see Table 2).

**Table 1:** Demographic details of cases and controls.

	**Cases**		**Controls**	
	**Young**	**Old**	**Young**	**Old**
*N*	5	5	5	5
Male/female [*n*]	3/2	3/2	3/2	3/2
**Age**				
Mean	56.0	93.6	56.4	92.6
Median	57	93	58	92
Range	52–59	91–99	51–59	90–99

**Table 2:** Clinical details of cases and controls.

**Status**	**Age [years]**	**Sex**	**Place of death**	**PMI [days]**	**Key medical history**	**Symptom duration until death [days]**	**Microbiological findings [cycle threshold; viral load/10^**6**^]**	**Cause of death**	**Key microscopic findings**
Case #1	99	M	Nursery home	2	Parkinsonism	4	24.99; 0.324	Pneumonia	Corticobasal degeneration
Case #2	93	M	Hospital	3	%	14	22.9; 1.43	Pneumonia	Alzheimer's disease; cerebral amyloidangioathy
Case #3	93	M	Hospital	1	%	n.a.	18.6; 30	Pneumonia	Alzheimer's disease; ARTAG
Case #4	92	F	Nursery home	2	Pulmonary hypertension	30	18.37; 35.3	Pneumonia	Alzheimer's disease
Case #5	91	F	Nursery home	2	Dementia	9	33.74; <0.01	Pneumonia	Alzheimer's disease; cerebral amyloidangioathy
Case #6	59	F	At home	5	%	n.a.	27.9; 412	Pneumonia	None
Case #7	58	M	At home	Unknown	Alcohol abuse, adipositas	n.a.	20.76; 6.49	Unclear (DVT, no LAE; no pneumonia)	None
Case #8	57	M	Hospital	5	Arterial hypertension, adipositas	22	23.73; 79.2	Pneumonia	None
Case #9	54	F	Hospital	1	Adipositas	5	32.06; 2160	Pneumonia	None
Case #10	52	M	At home	1	Adipositas	10	31.44; 3360	DVT and LAE	None
Control #1	99	M	At home	15	COPD	%	%	Upper gastrointestinal bleeding	Alzheimer's disease
Control #2	92	M	Hospital	13	Cardiac insufficiency	%	%	Cardiac failure	Alzheimer's disease
Control #3	92	M	Nursery home	5	Prostatic carcinoma; colonic carcinoma	%	%	Pneumonia	Alzheimer's disease
Control #4	90	F	Hospital	8	Fall to the ground; anticoagulation	%	%	Hemorrhagic shock	Alzheimer's disease; cerebral amyloid angiopathy
Control #5	90	F	Hospital	8	Myocardial infarction	%	%	Cardiogenic shock	Alzheimer's disease
Control #6	59	M	Hospital	3	Myocardial infarction	%	%	Cardiogenic shock	None
Control #7	59	M	Hospital	8	Syncope; diarrhea	%	%	Upper gastrointestinal bleeding	None
Control #8	58	F	Hospital	8	Pneumonia; dialysis	%	%	Cardiac failure	None
Control #9	55	F	Hospital	11	Hodgkin's disease	%	%	Hepatic failure	None
Control #10	51	M	Hospital	3	Adipositas; cardiomyopathy	%	%	Cardiac failure	None

*PMI, postmortem interval; DVT, deep venous thrombosis; LAE, lung artery embolism; ARTAG, Aging-related tau astrogliopathy; %, not available; n.a., not applicable*.

### Young COVID-19 Patients Show a Significant Microglia Activation in Frontal Cortex and White Matter

**Figure 2:**
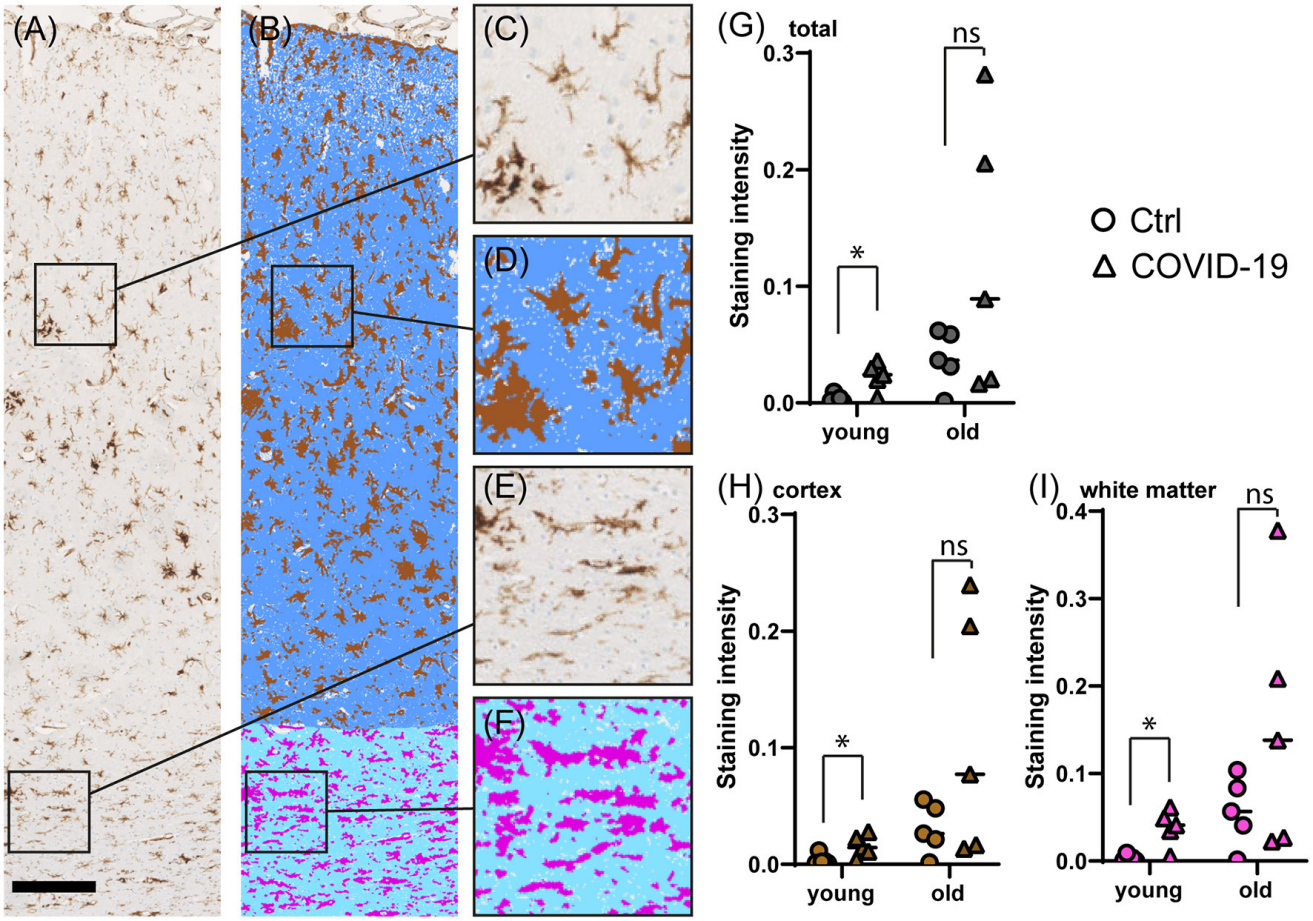
Comparison of microglial marker HLA immunostaining in COVID-19- and control cerebral tissue stratified according to patient age at death. **(A–F)** Representative images of cerebral HLA immunostaining **(A)**, comprising cortex **(C)** and white matter **(E)**. The colorized overlay image **(B)** indicates categorized pixel intensities in the cortex [**(D)**, brown: DAB, indigo: immunonegative tissue] and white matter [**(F)**, magenta: DAB, sky blue: immunonegative tissue]. Scale bar is 250 μm. **(G–I)** Dot plots showing staining intensity ratios calculated from cerebral tissue in young (<60y) vs. old (≥90y) patients in total **(G)**, and separately in the cortex **(H)** and white matter **(I)**. *p < 0.05, ns, not significant; *n* = 5 per group.

**Figure 3:**
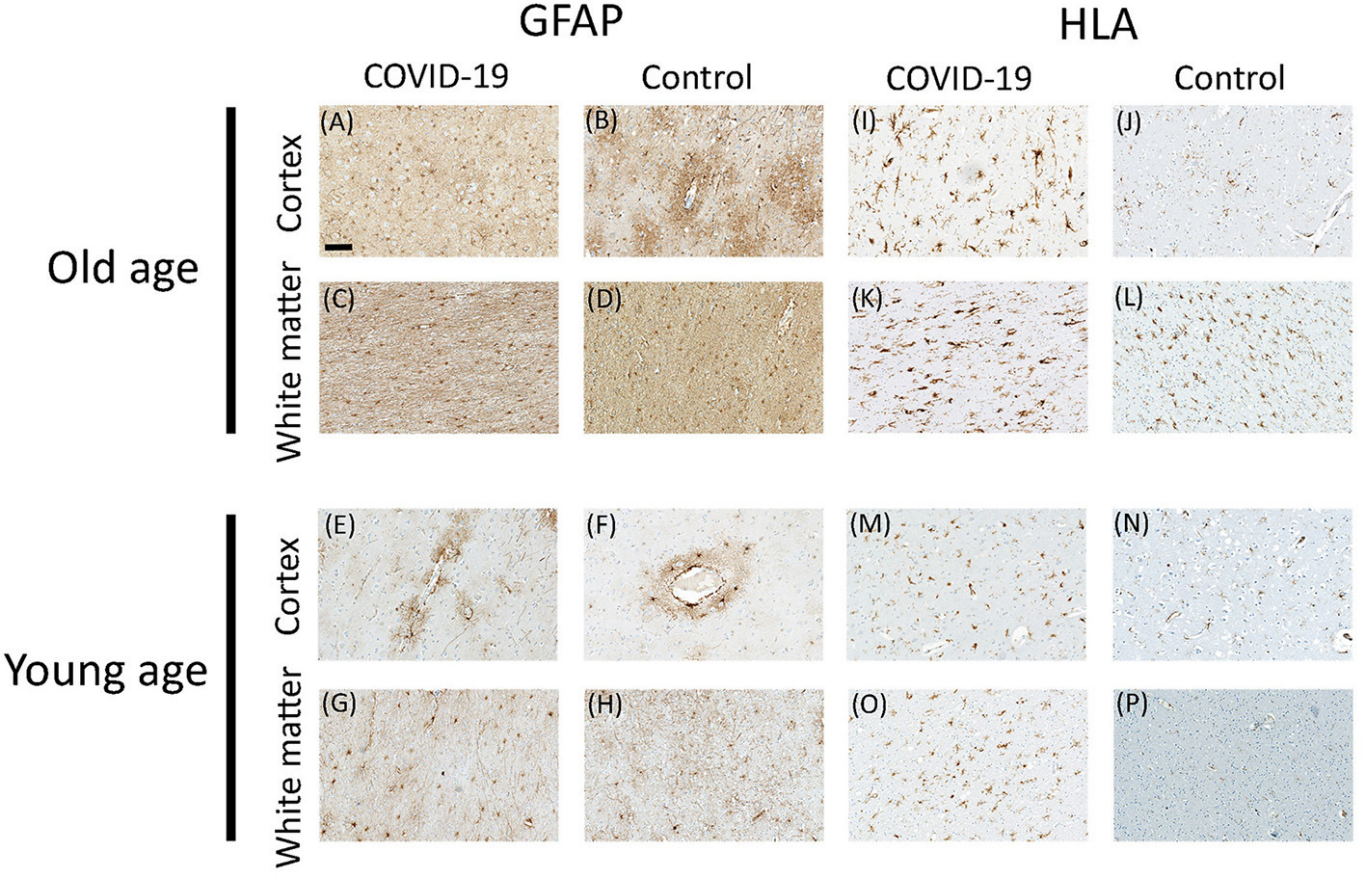
Representative microscopic images for GFAP and HLA immunostaining in COVID-19 patients and controls. GFAP staining in cortex **(A,B)** and white matter **(C,D)** of old COVID-19 patients and controls; and GFAP staining in cortex **(E,F)** and white matter **(G,H)** of young COVID-19 patients and controls. HLA staining in cortex **(I,J)** and white matter **(K,L)** of old COVID-19 patients and controls; and HLA staining in cortex **(M,N)** and white matter **(O,P)** of young COVID-19 patients and controls. Scale bar = 100 μm.

We performed immunostaining for HLA-DR, DP, DQ (HLA) as a marker of activated microglia (17), and quantified staining intensities *via* digital image analysis. When comparing brain tissue of young COVID-19 patients to young control patient we saw significantly increased microglia activation both in the frontal cortex and in the frontal white matter (mean HLA total: 0.02 vs. 0.004; *p* = 0.016; mean HLA cortex: 0.04 vs. 0.004; *p* = 0.016; mean HLA white matter: 0.02 vs. 0.004; *p* = 0.032; see Table 3, Figure 2). The difference was less and non-significant for old COVID-19 patients in comparison with old controls (mean HLA total: 0.12 vs. 0.04; *p* = 0.421; mean HLA cortex: 0.15 vs. 0.06; *p* = 0.421; mean HLA white matter: 0.11 vs. 0.03; *p* = 0.421). Moreover, comparisons across both age groups rendered insignificant differences in microglial activation between COVID-19 and control patients (mean HLA total: 0.07 vs. 0.02; *p* = 0.105; mean HLA cortex: 0.10 vs. 0.03; *p* = 0.063; mean HLA white matter: 0.06 vs. 0.02; *p* = 0.089; see also Figure 3).

**Table 3:** Results of quantitative image analysis of GFAP- and HLA-DR-staining of COVID-19 patients and controls (see text for details).

		**All ages**			**Old age**			**Young age**		
**Parameter**		**Case**	**Control**	***p*** **-value**	**Case**	**Control**	***p*** **-value**	**Case**	**Control**	***p*** **-value**
**GFAP**	Total	0.11	0.14	0.529	0.16	0.20	0.548	0.07	0.08	0.421
	Cortex	0.09	0.15	0.353	0.15	0.21	0.421	0.04	0.08	0.548
	Cortex perivascular	0.11	0.15	0.436	0.17	0.22	0.548	0.04	0.09	0.548
	Cortex non-perivascular	0.09	0.14	0.353	0.14	0.21	0.421	0.04	0.08	0.548
	White matter	0.13	0.15	0.684	0.15	0.19	0.421	0.11	0.10	0.690
	White matter perivascular	0.14	0.16	0.436	0.16	0.20	0.421	0.12	0.13	0.548
	White matter non perivascular	0.13	0.14	0.684	0.15	0.19	0.421	0.10	0.09	0.841
**HLA-DR**	Total	0.07	0.02	0.105	0.12	0.04	0.421	0.02	0.004	0.016
	Cortex	0.10	0.03	0.063	0.15	0.06	0.421	0.04	0.004	0.016
	White matter	0.06	0.02	0.089	0.11	0.03	0.421	0.02	0.004	0.032

### No Difference in Reactive Astrogliosis Between COVID-19 Patients and Controls

**Figure 4:**
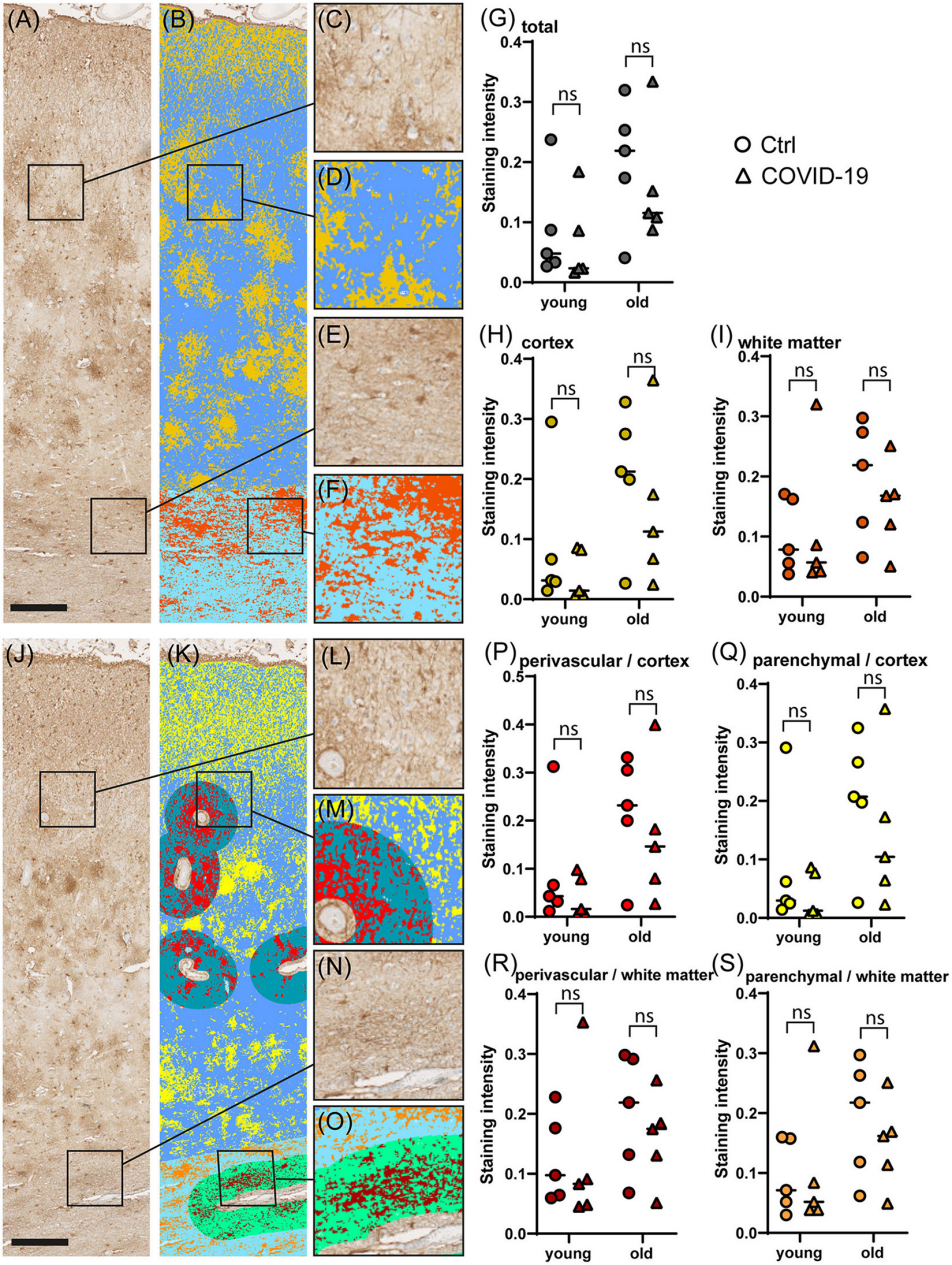
Comparison of GFAP immunostaining in COVID-19 and control cerebral tissue stratified according to patient age. **(A–F)** Representative images of cerebral GFAP immunostaining **(A)**, comprising cortex **(C)** and white matter **(E)**. The colorized overlay image **(B)** indicates categorized pixel intensities in the cortex [**(D)**, gold: DAB, blue: immunonegative tissue] and white matter [**(F)**, orange: DAB, sky blue: immunonegative tissue]. Scale bar is 250 μm. **(G–I)** Dot plots showing staining intensity ratios calculated from cerebral tissue in young (<60y) vs. old (≥90y) patients in total **(G)**, and separately in the cortex **(H)** and white matter **(I)**. **(J–O)** Representative image of cerebral GFAP immunostaining **(J)**, comprising cortex **(L)** and white matter **(N)**. The colorized overlay image **(K)** indicates categorized pixel intensities in perivascular regions of the cortex [**(M)**, red: DAB, turquoise: immunonegative tissue] and parenchymal regions of the cortex [**(M)**, yellow: DAB, blue: immunonegative tissue] as well as perivascular regions of the white matter [**(O)**, mocha: DAB, light green: immunonegative tissue] and parenchymal regions of the white matter [**(O)**, orange: DAB, sky blue: immunonegative tissue]. Scale bar is 250 μm. **(P–S)** Dot plots showing staining intensity ratios in young (<60y) vs. old (≥90y) patients in cortical perivascular **(P)** and cortical parenchymal **(Q)** as well as perivascular white matter **(R)** and parenchymal white matter **(S)** regions. *p < 0.05, ns, not significant; *n* = 5 per group.

**Figure 5:**
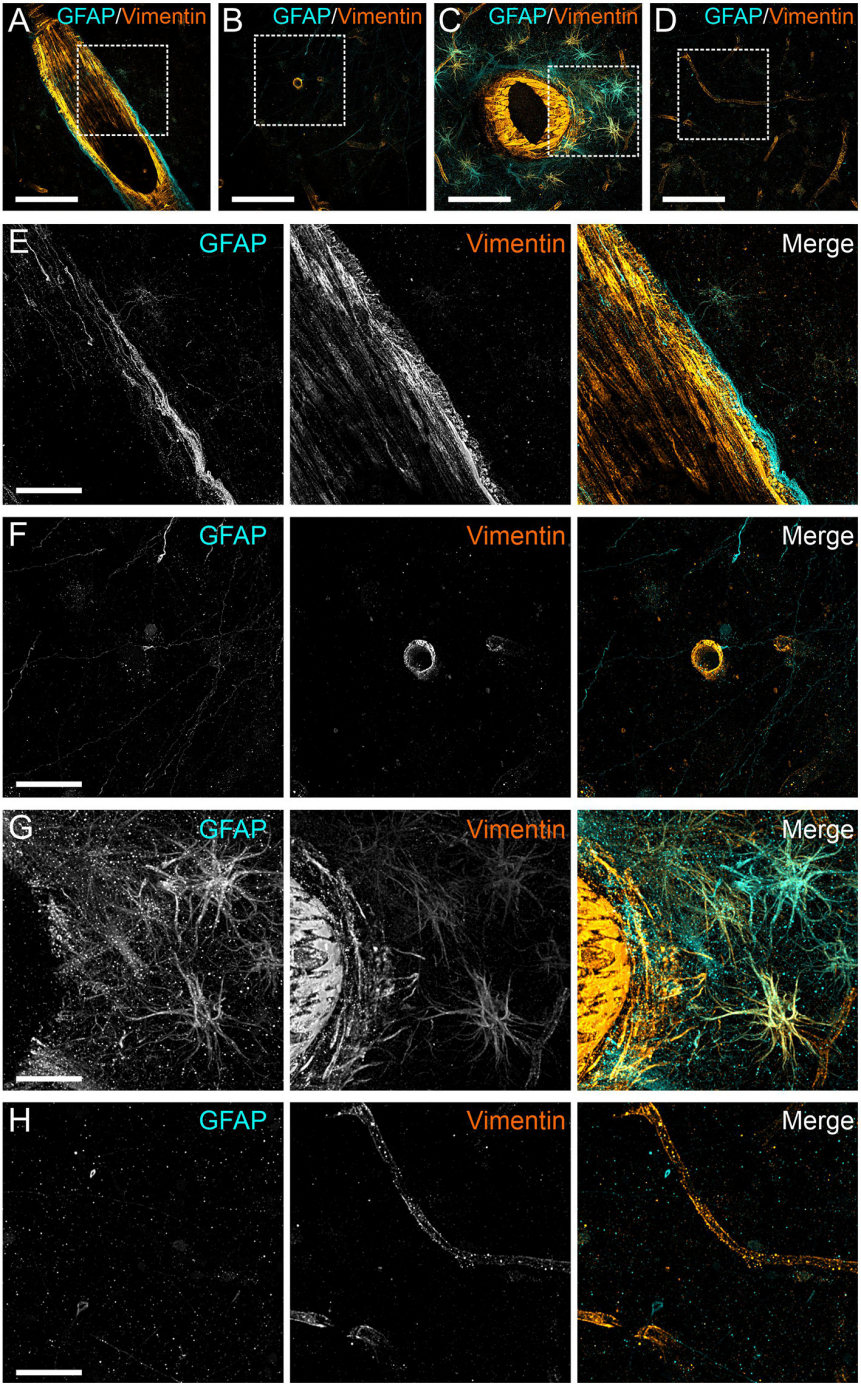
Anti-GFAP and anti-vimentin immunoreactivity of human cortical gray matter in COVID-19 patients. Three-dimensional projection of vimentin (orange) and GFAP (cyan) antibody staining of 250 μm thick sections of perivascular **(A)** and parenchymal **(B)** gray matter of frontal cortex of a young patient with COVID-19 (age 54). Perivascular **(C)** and parenchymal **(D)** staining of an elderly patient (age 99). Squares indicate regions depicted in the following images. **(E–H)** magnified views of the stainings shown in **(A–D)**. Scale bars: **(A–D)**, 100 μm; **(E,F)**, 30 μm.

To determine the degree of astrocyte activation, brain tissue was stained with the pan-astrocyte activation marker GFAP. Given the well-known close interplay of astrocytes and blood vessels, we separately subjected perivascular and parenchymal compartments of both cortex and white matter to immunostaining quantification. Analysis of staining intensities were performed as described above. Statistical comparisons concerning the degree of reactive astrogliosis in frontal cortex and frontal white matter showed an age-dependent increase but no significant differences between COVID-19 patients and controls neither of all ages nor when analyzed for both age groups separately or when comparing different anatomical regions (see Table 3, Figures 3, 4). In addition, there was no correlation for the degree of reactive astrogliosis and the disease duration (data not shown). Immunofluorescence staining of 200–250 μm free-floating sections further confirmed the results of the conventional immunohistochemical staining revealing a much larger number of astrocytes with intense GFAP labeling in old COVID-19 patients compared to young COVID-19 patients (see Figure 5, Supplementary Movie 1). Vimentin immunostaining used to visualize endothelial cells and reactive astrocytes highlighted perivascular astrocytes only in old COVID-19 patients (see Figure 5, Supplementary Movie 1), while parenchymal astrocytes in both old and young COVID-19 patients were vimentin-negative.

### No Difference in Cytotoxic T Lymphocyte Infiltration Between COVID-19 Patients and Controls

After immunostaining for CD8 and determining the semiquantitative score as describe above, further analysis revealed no significant difference in cytotoxic T lymphocyte infiltration in frontal cortex and frontal white matter between COVID-19 patients and controls neither for all ages (cortex: *p* = 0.574; white matter: 0.494; χ^2^ test) nor when analyzed for younger (cortex: *p* = 0.549; white matter: 0.565; χ^2^ test) or older age separately (cortex: *p* = 0.527; white matter: 0.135; χ^2^ test; see also Table 4).

**Table 4:** Semiquantitative analysis of CD8-positive T-lymphocytes in COVID-19 patients and controls.

		**Score**	**All ages**		**Old age**		**Young age**	
**Parameter**			**Case (%)**	**Control (%)**	**Case (%)**	**Control (%)**	**Case (%)**	**Control (%)**
								
**CD8**	Cortex	None	5 (50)	5 (50)	2 (40)	3 (60)	3 (60)	2 (40)
		Mild	5 (50)	4 (40)	3 (60)	2 (40)	2 (40)	2 (40)
		Moderate	0 (0)	1 (10)	0 (0)	0 (0)	0 (0)	1 (20)
		Severe	0 (0)	0 (0)	0 (0)	0 (0)	0 (0)	0 (0)
	White matter	None	1 (10)	3 (30)	0 (0)	2 (40)	1 (20)	1 (20)
		Mild	7 (70)	6 (60)	3 (60)	3 (60)	4 (80)	3 (60)
		Moderate	2 (20)	1 (10)	2 (40)	0 (0)	0 (0)	1 (20)
		Severe	0 (0)	0 (0)	0 (0)	0 (0)	0 (0)	0 (0)

### No Immunohistochemical Positivity for SARS-CoV-2 in COVID-19 Patients

After immunohistochemistry for SARS-CoV-2 nucleocapsid, none of all 10 COVID-19 patients showed any positivity in all sections investigated (data not shown).

## Discussion

Although coronavirus disease 19 (COVID-19) is considered mainly a respiratory disease, it is also the cause of a wide variety of neurologic symptoms during the acute phase of the disease. In addition, a significant percentage of survivors continue to suffer from fatigue-like symptoms, neurocognitive problems, depression, and other long-lasting symptoms. As of today, the exact causes of this illness, known as long COVID or post-acute sequelae of SARS-CoV-2 (PASC), are not known (4). In addition, it has been speculated that infection with SARS-CoV-2 might lead to hitherto unknown neurological ailments, akin to encephalitis lethargica in the aftermath of the 1918 influenza pandemic (24, 25). Therefore, an understanding of the pathophysiological mechanisms behind both acute and chronic effects of SARS-CoV-2 to the nervous systems is in urgent need stressing the importance of systematic neuropathological studies. Of note, even younger patients with only mild acute COVID-19 are prone to develop long COVID, thus raising concerns of a potential major public health and economic crisis.

A main question is whether neuropsychiatric complications in COVID-19 are due to direct virus infection of brain cells or whether they might rather be induced by secondary host effects such as an overshooting immune response (26). The determination of the respective pathways might have direct impact on subsequent potential therapeutic approaches.

In the present study we compared both older and younger COVID-19 patients with age-matched controls to further elucidate the possible pathophysiological mechanisms behind the neuropsychiatric complications following infection with SARS-CoV-2. Since it is well known that patients with other severe, non-SARS-CoV-2-related illnesses that have led to a stay in an intensive care unit with mechanical ventilation and other invasive measures can also develop neuropsychiatric symptoms not dissimilar to long COVID (27, 28), we restricted our analysis to patients who had not been treated in an intensive care unit and who did not show any clinical or pathomorphological signs of sepsis.

As the COVID-19 pandemic lingers on, our understanding of potential routes of infection to the nervous system has considerably enlarged. It has become increasingly clear that SARS-CoV-2 does not or at least not to a relevant amount access to the brain *via* olfactory infection, as had been surmised during the early phase of the pandemic (29, 30). Alternatively, the virus seemingly enters the brain through the blood-brain-barrier by using infection of endothelial cells as an intermediate station or hijacking leucocytes as Trojan horses into the brain parenchyma (9, 31, 32). Having reached the brain, the virus binds to angiotensin-converting enzyme 2 (ACE-2) receptors that are widely expressed in the CNS and are regulated by nicotinic receptors (33, 34). Irrespective of the exact route, it has gradually emerged that the main culprit of CNS damage in COVID-19 patients is probably not related to damaging SARS-CoV-2 neurotropism but rather to an increase of innate immune activation and / or simmering inflammation which puzzlingly takes place without the presence of detectable virus particles (35). Infection with SARS-CoV-2 leads to a rise in levels of peripheral cytokines in the blood which in turn may activate microglia and neurotoxic astrocytes (36, 37). However, so far astrogliosis in COVID-19 has not been studied in much detail. Single nucleus transcriptional analyses of brain cells in patients who had died of COVID-19 revealed that astrocytes indeed acquire a specific transcriptional profile characterized by an up-regulation of interferon-gamma signaling (8). Interestingly, in that study an increase of the pan-astrocyte marker GFAP on the transcriptional level was detected. In contrast, in our study the amount of GFAP protein levels by immunohistochemistry obviously increased with age but remained largely unaffected by infection with SARS-CoV-2. However, since the brain regions investigated by transcriptional analysis of isolated nuclei in the study by Yang et al. (8) and by immunohistochemistry in our current study are not identical, the examination of other brain regions–preferably involving both transcriptional and immunohistochemical assessment–would certainly be interesting in the future.

Since astrocytes also build up parts of the blood-brain barrier (BBB), we were specifically interested if we could detect any regional increase in the abundance of GFAP around brain vessels. The BBB might serve as a potential entry point of SARS-CoV-2 into the brain (7, 9). Moreover, immune activation including specific T-cell cluster affecting the brain vasculature has been detected in brains of COVID-19 patients (7). Interestingly, GFAP staining intensity was not increased in the perivascular compartment in COVID-19 patients in our study. Future studies might help to determine if subsets of astrocytes around blood vessels might adopt a specific pro-inflammatory phenotype, such as the recently described neurotoxic A1 reactive astrocytes (38).

Although reports on the presence of SARS-CoV-2 in the brain differ considerably, we only detected single infected cells in the brain in a subset of patients by immunohistochemistry in a recent post-mortem case series (12), while in this cohort none showed any positivity. However, the failure of detecting viral particles in the brain should not be used as an argument for the absence of SARS-CoV-2-related tissue pathology, since this may be due to transient infection of the brain during early phases of the disease (35). Recent studies therefore started to also include patients in the acute phase of COVID-19 disease (39). In line with most recent findings, in our set of COVID-19 patients none showed evidence of direct SARS-CoV-2 neuroinvasion when assessed by immunohistochemistry, but a high degree of innate immune activation related to microglia (40). Furthermore, we could show that microglia activation is more pronounced in younger patients. In comparison, the degree of microglial activation in older patients was more variable with some COVID-19 patients showing a very high intensity of HLA-DR staining (see Figure 2). This is in line with other studies showing a similar variability in the degree of neuroinflammation while simultaneously demonstrating anatomically compartmentalized immune activation especially in perivascular regions accompanied by infiltration of CD8 and CD4 T-cells (7). These findings argue in favor of the theory that SARS-CoV-2 may enter the parenchyma *via* the BBB (7, 9), although secondary alterations of the BBB function due to systemic effects of virus infection cannot be excluded.

Our study has limitations. Firstly, the number of patients and controls is relatively small; therefore, our results are of a preliminary nature, stressing the need for further extensive analysis of larger cohorts with additional antibodies to further characterize microglial phenotypes (17). Secondly, for now we concentrated on the analysis of the frontal cortex; acknowledging that the astroglial and microglial response might differ between brain regions, the study of more regions should be appropriate. Thirdly, investigation with different markers for microglia (e.g., TMEM119, Iba-1) and/or astrocytes might show different results. Lastly, being drawn from an autopsy study, our findings necessarily represent only the most severe end of the COVID-19 spectrum and thus are probably not generalizable to milder cases.

## Data Availability Statement

The raw data supporting the conclusions of this article will be made available by the authors, without undue reservation.

## Ethics Statement

The studies involving human participants were reviewed and approved by Ethics Committee of the Hamburg Chamber of Physicians, Hamburg, Germany. Written informed consent for participation was not required for this study in accordance with the national legislation and the institutional requirements.

## Author Contributions

JM, HL, MD, and MG contributed to conception and design of the study. JM and MD performed the statistical analysis. MD and HL performed the quantitative image analysis. JM wrote the first draft of the paper. CL, MB, NR, KS, and SP wrote sections of the manuscript. BO, AF, J-PS, and SP provided acquisition and interpretation of data. SK, HA, GG, CL, MB, KS, NR, and SP contributed substantially to the conception and design of the project. All authors contributed to manuscript revision, read, and approved the submitted version.

## Funding

This study was funded by Defeat Pandemics (01KX2021), NATON (01KX2121) within the Network University Medicine (German Federal Ministry of Education and Research, BMBF, and Deutsche Forschungsgemeinschaft (SFB 1328, project number 335447717).

## Conflict of Interest

The authors declare that the research was conducted in the absence of any commercial or financial relationships that could be construed as a potential conflict of interest.

## Publisher's Note

All claims expressed in this article are solely those of the authors and do not necessarily represent those of their affiliated organizations, or those of the publisher, the editors and the reviewers. Any product that may be evaluated in this article, or claim that may be made by its manufacturer, is not guaranteed or endorsed by the publisher.
